# IL-33 Modulates Cytotoxic NK Cell Subsets in Severe Eosinophilic Asthma

**DOI:** 10.3390/life16020348

**Published:** 2026-02-18

**Authors:** Laura Bergantini, Irene Paggi, Tommaso Pianigiani, Elena Bargagli, Paolo Cameli

**Affiliations:** Respiratory Disease Unit, Department of Medical Sciences, University Hospital of Siena (Azienda Ospedaliera Universitaria Senese, AOUS), Viale Bracci, 53100 Siena, Italy

**Keywords:** NK cells, IL-33, severe eosinophilic asthmatic patients

## Abstract

Natural Killer (NK) cells contribute to airway inflammation in severe eosinophilic asthma (SEA). IL-33, elevated in SEA, may modulate NK cell function, but its effects are unclear. We analyzed peripheral blood NK cell subsets from five SEA patients and five healthy controls using flow cytometry, assessing CD56/CD16-defined subsets and markers, CD57, NKG2A, CD62L, and ICAM-1, at baseline and after 72 h IL-33 stimulation. SEA patients showed reduced mature cytotoxic NK cells and altered expression of adhesion and regulatory molecules. IL-33 selectively increased ICAM-1 and NKG2A in mature NK cells, while decreasing these markers in immature subsets. These findings indicate that IL-33 differentially regulates NK-cell phenotype and function, highlighting NK cells as dynamic mediators of inflammation in SEA.

## 1. Introduction

In recent years, Natural Killer (NK) cells have emerged as important contributors to the immunopathogenesis and progression of severe asthma (SA). Beyond their classical role in innate immune surveillance and antiviral defence, NK cells have been increasingly implicated in the regulation of airway inflammation and immune polarization in asthmatic disease. Several studies have demonstrated that NK cells may actively promote type 2 immune responses, contributing to the development and maintenance of eosinophilic inflammation and airway hyperresponsiveness, hallmarks of severe asthma [1,2,3].

Alterations in NK cell function have been specifically associated with severe forms of the disease. Duvall and colleagues reported that the immunological environment of the SA airway is characterized by a marked reduction in NK cell cytotoxic activity, a dysfunction that appears to be further exacerbated by corticosteroid treatment. Since corticosteroids remain a cornerstone of asthma therapy, their inhibitory effects on NK cell effector functions may paradoxically contribute to persistent airway inflammation and impaired immune regulation in severe asthma [4,5]. These findings suggest that qualitative and quantitative alterations in NK cells may play a critical role in sustaining chronic inflammation and disease severity.

Among the epithelial-derived cytokines involved in asthma pathogenesis, interleukin-33 (IL-33) has been extensively investigated. IL-33 is rapidly released upon epithelial injury and acts as an alarmin, initiating and amplifying immune responses by orchestrating the recruitment and activation of multiple immune cell types involved in asthma symptoms [6]. In asthmatic patients, IL-33 has been shown to enhance the production of type 2 cytokines, thereby promoting eosinophilic inflammation and airway remodelling [7]. However, accumulating evidence indicates that the immunological effects of IL-33 are highly context-dependent. In healthy individuals, IL-33 exposure has been associated with a predominantly type 1 immune profile, characterized by interferon-gamma production, largely attributed to NK cells [8].

Despite these observations, the role of IL-33 in modulating NK cell phenotypes and functional subsets within the context of asthma remains poorly understood. It is unclear whether IL-33 differentially affects NK cell subsets in severe eosinophilic asthma compared to healthy conditions, and how this modulation may contribute to disease persistence or progression.

In this study, we first aimed to characterize differences in NK cell surface markers between healthy controls (HC) and patients with severe eosinophilic asthma (SEA) who are eligible for monoclonal antibody treatments. Furthermore, we investigated the effects of IL-33 stimulation on NK cell subsets in peripheral blood from SEA patients and HC to assess the IL-33-driven modulation of NK cell phenotypes. By elucidating the interaction between IL-33 signalling and NK cell biology in severe asthma, this work seeks to provide new insights into the immunological mechanisms underlying disease severity and potential therapeutic targets.

## 2. Materials and Methods

### 2.1. Study Population

We retrospectively included 5 well-characterized SEA patients and 5 healthy controls (HC), which were age- and sex-matched. Eligible participants were untreated with oral corticosteroid for almost 3 months to avoid immunological alterations. Written informed consent was obtained after institutional review board CEAVSE approval (BE-ACTIVE prot. n. 21210, date: 13 December 2021). Given the observational nature of this translational study, there was no randomization or formal blinding process for the investigator.

### 2.2. Peripheral Blood Mononuclear Cells Collection

**Figure 1:**
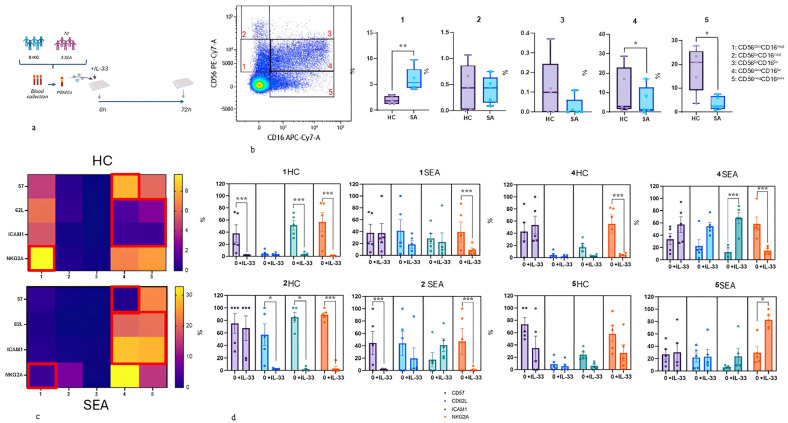
Natural killer (NK) cell subsets and IL-33-induced modulation in healthy controls (HC) and severe eosinophilic asthma (SEA). (**a**) Study workflow: PBMCs from HC (n = 5) and SEA (n = 5) were collected and cultured for 72 h with or without IL-33. (**b**) Gating strategy for identification of five NK-cell subsets based on CD56/CD16 expression and comparison of subset frequencies between HC and SEA (* *p* < 0.05; ** *p* < 0.01). (**c**) Baseline heatmaps showing expression of CD57, CD62L, ICAM-1 and NKG2A across NK cell subsets in HC and SEA. (**d**) Expression of the same markers in NK cell subsets following IL-33 stimulation. Statistical significance indicated as * *p* < 0.05; *** *p* < 0.001.

Peripheral blood samples (PB) were collected from HC and from SEA patients. Cells from PB were analyzed through multiparametric flow cytometry (BD FacsLyrics, NJ, USA). The study design is reported in Figure 1a. Peripheral blood mononuclear cells were separated in the laboratory of the Respiratory Diseases Unit, Siena University Hospital (Italy) in the period January 2019 to December 2022. Peripheral blood samples were drawn into a tube containing EDTA anticoagulant (BD Vacutainer^®^ EDTA tubes, BD Biosciences, CA, USA) and processed within 8 h. PBMCs were then separated by gradient centrifuging (Ficoll Histopaque^®^-1077, Sigma-Aldrich), washed twice, resuspended in 80% RPMI 1640, 10% FBS and 10% dimethyl sulfoxide (DMSO) at 2 × 10^6^ cells per vial, and stored in liquid nitrogen until analysis.

### 2.3. PBMCs Stimulation and Flow Cytometric Analysis

PBMCs were stimulated with IL-33 (0.1 mg/mL) [9,10] for 72 h. NK cells were identified after the exclusion of doublets, dead cells, and CD19^+^CD14^+^CD3^+^ cells. Four different NK cell subsets were selected, as reported in Figure 1b, based on the expression of CD56 and CD16 (Figure 1b). In these subsets, the expression of CD57, NKG2A, CD62L, and ICAM-1 was explored.

### 2.4. Statistical Analysis

Data in the graphs are expressed as means ± SD. The statistical significance of differences in parametric data was assessed by the two-tailed Student’s *t* test or one-way analysis of variance. Differences in nonparametric data between groups were assessed by the Wilcoxon matched-pairs signed-rank test or the Kruskal–Wallis with post hoc test with correction for multiple comparisons by Dunn’s test. A *p*-value < 0.05 was considered significant. Prism 10.2. (GraphPad) was used to analyze the data.

## 3. Results

### 3.1. Clinical Features of Study Population

The main characteristics of our study population are reported in Table 1. No differences in the distribution of sex, age or smoking habits emerged between HC and SEA patients. SEA patients showed poor control of asthma at T0, as assessed by ACT and ACQ-7, associated with a high annualized exacerbation rate.

### 3.2. NK Cells Behaviour

As reported in Figure 1b, a decreased percentage of the most mature CD56^dim^CD16^br^ and CD56^neg^CD16^+^ NK cell phenotypes was observed. Additionally, a decrease in the most immature CD56^dim^CD16^neg^ NK cells was reported. In SEA patients, an increased expression of adhesion molecules, including CD62L and ICAM-1, was observed in mature NK cells phenotypes (CD56^dim^CD16^br^, and CD56^neg^CD16^pos^). A decreased expression of CD57 was detected in CD56^dim^CD16^br^ NK cells in SEA patients (Figure 1c).

By analyzing the behaviour of these markers after 3 days of stimulation with IL-33, the cytotoxic phenotypes of NK (CD56^dim^CD16^br^) showed an increase in ICAM-1 expression, which markedly declined in the other (more immature) NK cells (CD56^br^CD16^neg^).

Concomitantly, IL-33 was found to modulate NKG2A expression in all NK cell subsets, particularly the most mature phenotypes, CD56^neg^CD16^pos^ (Figure 1d).

CD56^dim^CD16^br^ also showed a trend toward increased expression, although without reach statistical significance (Figure 1d). The opposite behaviour was also reported for CD57, which showed increased expression in mature NK cell subsets of HC and decreased in the most immature NK cells of SEA patients following IL-33 stimulation (Figure 1d).

## 4. Discussion

In this study, we support previous evidence that SEA patients are characterized by profound alterations in NK cell phenotype and responsiveness, particularly within the most mature cytotoxic subsets [5]. SEA patients exhibited reduced frequencies of CD56^dim^CD16^br^ accompanied by altered expression of key regulatory and adhesion molecules, including ICAM-1, CD62L, CD57, and NKG2A. Notably, IL-33 stimulation further modulated these markers in a subset-specific manner, enhancing ICAM-1 and NKG2A expression predominantly in mature NK cell populations while exerting opposite effects on immature subsets. These findings suggest that IL-33 may differentially shape NK cell functionality in SEA, potentially influencing their cytotoxic potential, migratory behaviour, and regulatory balance.

Earlier studies demonstrated that IL-33 can directly activate NK cells, enhance cytokine production, and modulate inhibitory and activating receptor expression, particularly in the context of type 2 inflammation and allergic disease [11,12,13]. Our data are consistent with these findings and extend the current knowledge by providing a detailed phenotypic analysis of NK cell subsets in severe eosinophilic asthma patients. We show that IL-33 differentially modulates adhesion molecules (CD62L, ICAM-1) and maturation markers (CD57) across distinct NK cell subsets, highlighting a phenotype-dependent responsiveness to IL-33 stimulation.

ICAM-1 has been shown to play a key role in the pathogenesis of asthma where, in addition to helping the recruitment of T cells into inflamed tissue, it is the key entry protein for viruses. Recently, it has been described that in tumour environments, its downregulation on NK cells is a mechanism for evading monoclonal antibody-mediated ADCC or CAR-induced NK cell cytotoxicity [14].

NKG2A is an inhibitory receptor on NK cells that has established regulatory functions in the direct interaction with target cells when engaged with its ligand, the non-classical HLA class I molecule HLA-E. NKG2A maintains NK cell expansion capacity by dampening both proliferative activity and excessive activation-induced cell death [15]. Interestingly, CD62L NK cells are characterized by the ability to combine the production of interferon-gamma and in vivo proliferation during viral infection, with the capacity to kill and produce cytokines via engagement with activating receptors. Both adhesion molecules, ICAM-1 and CD62L, were shown to be altered during the acute exacerbation of severe asthmatic patients. NK cells expressing ICAM-1 and L-selectin may selectively migrate into inflamed lung tissues [16]. CD57^+^ NK cells are highly mature and terminally differentiated cells with a higher cytotoxic capacity, decreased capacity to proliferate and a decreased responsiveness to cytokines [17].

Overall, our data highlight NK cells as dynamic targets of IL-33 mediated immune regulation in SEA and support the hypothesis that dysregulated NK cell maturation and activation contribute to persistent airway inflammation. Moreover, it may be of particular interest to longitudinally monitor NK cell phenotypes in SEA patients undergoing monoclonal antibodies target therapy to determine whether these treatments contribute to the functional or phenotypic remodelling of NK cell subsets. Importantly, the distinct modulation observed in SEA compared with HC suggests that chronic exposure to IL-33 in severe asthma may contribute to NK cell dysregulation, potentially impacting immune surveillance and inflammatory amplification in the asthmatic airway [13]. Under these experimental conditions, the modulation of NK cell surface markers may also occur indirectly through soluble factors released by other IL-33-responsive cells, such as Th2 cells or basophils. This represents an inherent limitation of the experimental model and should be considered when interpreting the results. Further studies with larger cohorts are warranted to clarify the mechanistic consequences of these alterations and to determine whether modulation of NK cell pathways could represent a complementary therapeutic opportunity for patients with severe asthma. Another limitation of this study is that both the SEA and HC cohorts consisted exclusively of white patients. Therefore, the applicability of these findings to populations with different ethnic backgrounds requires validation in future studies including ethnically diverse cohorts.

## Figures and Tables

**Table 1:** Demographic characteristics of selected patients and HC. Results are expressed as means ± SD (range). Abbreviations: n/a, not applicable.

	Healthy Controls	Severe Eosinophilic Asthma
Number of subjects	5	5
Demographics		
Age (years)	56 ± 8	58 ± 13
Sex (% male)	40	40
Race (% white)	100	100
Smoking habits (% never smokers)	80	80
Asthma duration (years)	n/a	6.76 ± 15.77
Moderate–severe exacerbations/yrs	n/a	5.53 ± 1.39
ACT	n/a	12.6 ± 4.32
ACQ-7	n/a	10.5 ± 3.65

## Data Availability

Data can be made available upon request by the corresponding author.
